# The Medical Experts in the Van Ness-Wharton Trial

**Published:** 1873-08

**Authors:** 


### THE MEDICAL EXPERTS IN THE VAN NESS-WHARTON TRIAL.

We greatly fear that the medical profession has sustained a most serious, if not irreparable thrust in "the house of its friends," from those who, as far as we are able to judge, have violated some of the most conspicuous and generally admitted obligations of the profession. Whether this has been done with corrupt intent, as believed by some, we will not absolutely assert, but we feel constrained to present certain points of principle and propriety in connection with medical experts, which can scarcely be controverted, and, if true, reflecting most unfavorably upon Drs. Reese and Wood of Philadelphia, and perhaps others, as their conduct appears to us. We assert with perfect confidence that a medical expert has no right whatever to publish anything bearing upon the case in which he is engaged until such case has been finally and entirely disposed of by the courts. That whenever he writes at all, (even after the final adjudication of the case,) he has nothing to do with *circumstantial testimony*, but should confine himself strictly to the scientific points involved, he being neither advocate, judge, jury, nor *witness* in the ordinary sense. That he has, especially, no right to discuss the guilt or innocence of an alleged criminal previous to a *final* decision of the courts. That it is in the highest degree reprehensible to assail, at any time, the personal or professional character of opposing experts. That it is greatly to the discredit of medical men to introduce any personal matter whatever into the discussion of a scientific question. That medical experts should be specially careful to avoid everything which could by possibility subject them to the imputation of being the *advocates* of either party to a case. The individuals referred to stand charged with the violation of all these principles, and we regret to say with more serious offenses against truth and right. They stand charged, as we understand the matter, by gentlemen of the highest character, (with whom we are personally acquainted, and in whose intelligence and integrity of purpose we have the most unbounded confidence,) with manufacturing and perverting or suppressing testimony to meet the exigencies of their position, and with perverting the testimony that was given on the trial in order to maintain the view they attempted to establish. In addition to this it is also asserted upon the same authority that immediately after the close of the first trial, when it was known that Mrs. Wharton would be tried a second time, Reese wrote a review of the first trial, *as soon as it closed*, in which he made the most serious charges against Drs. Williams and others, the experts engaged by the prosecution, which paper, it seems was industriously and generally circulated in the locality where the trial was to take place. Then, it is stated upon the same authority, came the second trial—the jury disagreed, *eight* in favor of conviction and *four* in favor of acquittal, and everything indicating the certainty of another trial for the alleged attempt to murder Van Ness, when Wood immediately publishes a review of it in a literary magazine, and shortly afterward another in a medical journal, making the same attempt that Reese had made in the first trial to injure the medical witnesses for the State, and thus weaken their evidence in the next trial, and also, as is supposed, to create a public sentiment in favor of Mrs. Wharton. This is a most painful reference which we have felt ourselves, as journalists, constrained to make to a transaction which has unquestionably added to the disfigurement of American medicine, and could not have occurred, as we believe, except in accordance with the demoralizing influences incident to the fratricidal strife in which the country has so recently been engaged, the result of which has been the spawning of all manner of evils and the poisoning even of the very fountains of science. We can but hope that under the benign and purifying influences of peace, we shall gradually have a return to the old landmarks of virtue and honor in Government, and in every department of business, and especially within the sacred precincts of the science of life.

